# Host Genetic Factors Affecting Spontaneous HBsAg Seroclearance in Chronic Hepatitis B Patients

**DOI:** 10.1371/journal.pone.0053008

**Published:** 2013-01-11

**Authors:** Huei-Ru Cheng, Chun-Jen Liu, Tai-Chung Tseng, Tung-Hung Su, Hwai-I Yang, Chien-Jen Chen, Jia-Horng Kao

**Affiliations:** 1 Graduate Institute of Clinical Medicine, National Taiwan University, Taipei, Taiwan; 2 Department of Internal Medicine, National Taiwan University College of Medicine and National Taiwan University Hospital, Taipei, Taiwan; 3 Hepatitis Research Center, National Taiwan University College of Medicine and National Taiwan University Hospital, Taipei, Taiwan; 4 Department of Medical Research, National Taiwan University College of Medicine and National Taiwan University Hospital, Taipei, Taiwan; 5 Department of Internal Medicine, Buddhist Tzu Chi General Hospital Taipei Branch, Taipei, Taiwan; 6 Molecular and Genomic Epidemiology Center, China Medical University Hospital, Taichung, Taiwan; 7 Graduate Institute of Clinical Medical Science, China Medical University, Taichung, Taiwan; 8 Genomics Research Center, Academia Sinica, Taipei, Taiwan; Yonsei University College of Medicine, Republic of Korea

## Abstract

Spontaneous clearance of hepatitis B surface antigen (HBsAg) in chronic hepatitis B (CHB) patients usually indicates a remission of hepatitis activity and a favorable outcome. Two single nucleotide polymorphisms (SNP), rs3077 near HLA-DPA1 region and rs9277535 near HLA-DPB1 region, have been shown to be associated with HBV persistence after acute HBV infection. However, little is known about the impact of these 2 SNPs on spontaneous HBsAg clearance in CHB patients. In this case-control study, a total of 100 male HBeAg-negative chronic HBV carriers who cleared HBsAg spontaneously (case group) and 100 age-matched HBeAg-negative male patients with persistent HBsAg positivity (control group) were enrolled. We investigated the relationship between these 2 SNPs and HBsAg clearance. There was a higher frequency of rs9277535 non-GG genotype in the case group (57% vs. 42%). Patients with rs9277535 non-GG genotype had a higher chance to clear HBsAg [Odds ratio (OR): 1.83, 95% confidence interval (CI): 1.04∼3.21, P = 0.034]. Compared to GG haplotype of rs3077 and rs9277535, GA haplotype had a higher chance of achieving spontaneous HBsAg loss (OR: 2.17, 95% CI: 1.14∼4.16, P = 0.030). In conclusion, rs9277535 non-GG genotype is associated with a higher likelihood of spontaneous HBsAg seroclearance in CHB patients.

## Introduction

Hepatitis B virus (HBV) infection is a global health problem, resulting in over one million deaths per year [1]. Patients with chronic HBV infection are at risk of developing adverse outcomes, including cirrhosis and hepatocellular carcinoma (HCC), with an estimated lifetime risk of 25–40% [1]–[4].

Hepatitis B surface antigen (HBsAg) serves as important serological markers for the diagnosis and monitoring of chronic hepatitis B patients. Although spontaneous HBsAg loss is a rare event, it usually indicates a cure for the disease [5], [6]. Previous studies have indicated that HBsAg loss rate is around 0.5 to 1.4% per year in patients with chronic HBV infection [7]–[14]. Several host and viral factors, including older age, normal alanine aminotransferase (ALT) level, low serum levels of HBV DNA and HBsAg have been shown to be associated with a higher chance of HBsAg loss [6], [8], [11], [13]–[14]. However, little is known about whether host genetic factor influences spontaneous HBsAg loss in patients with chronic hepatitis B infection.

Single nucleotide polymorphisms (SNPs) represent a natural genetic variability at high density in the human genome. Recent study using genome-wide association studies (GWAS) have shown that two SNPs, rs3077 near HLA-DPA1 and rs9277535 near HLA-DPB1, are associated with persistence of HBV infection [15]. Since HLA-DPA1 and HLA-DPB1 code the functional subunit of major histocompatibility complex (MHC) class II [16], these data may suggest possible interactions between virus and host immunity. Taking these lines of evidence together, the aim of this study was to investigate whether viral and host genetic factors affect spontaneous HBsAg seroclearance in chronic HBV carrier in Taiwan.

## Materials and Methods

### Patient Cohort

We adopted an age-matched case-control study design. There were 100 HBeAg-negative HBV carriers who were positive for HBsAg at enrolment but cleared HBsAg during the average 6.3 years follow-up, which were designed as cases. Another 100 HBeAg-negative HBV carriers who remained HBsAg-positive during the whole course of average 8.9 years follow-up were designed as control. All of the 200 patients were all male and had no evidence of concomitant hepatitis C virus (HCV), hepatitis D virus (HDV) or human immunodeficiency virus (HIV) infection. For the 100 cases, 8 patients were enrolled from National Taiwan University Hospital and the other 92 patients were enrolled from the a Taiwanese community-based cohort study, Risk Evaluation of Viral Load Elevation and Associated Liver Disease/Cancer-Hepatitis B Virus (REVEAL-HBV) study [17]. The 100 age-matched controls were all enrolled from the National Taiwan University Hospital. For the case group, the clinical data and serum samples were collected when HBsAg seroclearance was achieved; for control group, the data and samples were collected at enrolment. Every enrolled patient signed the informed consent approved by the ethical committee for blood sampling.

### Definitions of sustained HBsAg seroclearance

HBsAg seroclearance was defined as HBsAg level measuring less than 0.05 IU/mL using the Architect HBsAg QT (Abbott Diagnostic, Germany) twice consecutively at least one year apart.

### Serological assays

Antibodies to HBsAg (anti-HBs), HBeAg, and anti-HBe were assayed with commercial kits (AxSYM System®, Abbott Laboratories, North Chicago, IL, USA). Antibody to HCV was tested by a second-generation enzyme-linked immunoassay (Abbott Laboratories). HBsAg level was quantified by the Architect HBsAg QT (Abbott Diagnostic, Wiesbaden, Germany) according to the manufacturer instructions [18]. The detection range of Architect assay is from 0.05 to 250 IU/mL. If the level was higher than 250 IU/mL, the samples were diluted to 1∶100 to 1∶1000 to obtain a reading within the range of the calibration curve.

### Quantification of serum HBV-DNA and HBV genotyping

Serum HBV-DNA level and HBV genotype were determined using real-time PCR-based single-tube assay as previously described [19]. Briefly, the method consists of two consecutive steps. The first step uses real-time PCR to quantify HBV-DNA, and the second step uses melting curve analysis to genotype HBV. The detection limit of HBV-DNA level is 20 IU/mL or 100 copies/mL. Because HBV genotype is presumed to be constant, available serum samples from HBeAg-positive stage were used to genotype HBV in patients with undetectable viral load at the indicated time point.

### Extraction genomic DNA

Genomic DNA was extracted from peripheral blood mononuclear cells by using the QIAamp kits (Qiagen, Inc., Valencia, CA, USA).

### Determination of single nucleotide polymorphisms of rs3077 and rs9277535

Genomic DNA samples from patients and controls were genotyped for the rs3077 and rs9277535 polymorphism with TaqMan SNP genotyping assays (Applied Biosystems Inc, Foster City, CA), using the StepOne Plus™ Real-Time PCR thermocycler, according to manufacturers recommended protocols. TaqMan probes and primers were designed and synthesized by Applied Biosystems Inc. Automated allele calling was performed using SDS software from Applied Biosystems Inc. Positive and negative controls were used in each genotyping assay.

### Ethical considerations

The study conformed to the ethical guidelines of the 1975 Declaration of Helsinki as reflected by a priori approval by the Institutional Review Board of the National Taiwan University Hospital. All patients gave their written informed consent at enrollment.

### Statistical analysis

Mean and standard deviation for continuous variables and percentages were used for categorical variables. For patients with undetectable serum HBV-DNA (copies/mL) and HBsAg (IU/mL), the lower limit of detection (20 IU/mL for HBV-DNA and 0.05 IU/mL for HBsAg) were assigned for analysis.

The statistical analysis was performed using STATA software (version 8.2; Stata Corp, College Station, TX, USA). All tests were 2-sided and a *p* value<0.05 was considered statistically significant. Regarding these 2 SNPs, a Hardy–Weinberg disequilibrium test was performed for each SNP [20]. Since rs3077 and rs9277535 are within *HLA-DP* region on chromosome 6 and 21839 base-pair apart, Linkage disequilibrium (LD) index (Lewontin's D′ and r^2^) index calculation and haplotype analysis was performed for both SNPs. LD index calculation was performed by Haploview version 4.1 software [21]. The OR of each haplotype was derived uisng UNPHASED statistical software version 3.1.4 [22].

## Results

### Baseline characteristics

We collected 100 male HBeAg-negative chronic hepatitis B patients who lost HBsAg spontaneously (case group) and 100 age-matched male patients with persistent HBsAg positivity (control group). In the case group, the duration from enrollment to HBsAg seroclearance was 6.3±4.1 years, while the average follow-up duration of control group was 8.9±4.8 years, which was longer than the case group (P = 0.001). In addition, the baseline characteristics are summarized in Table 1. Mean serum ALT level in the control group was higher than the case group (116.9 vs. 14.7 U/L, *P* = 0.0006). Mean logarithmic value of serum HBV DNA level were also significantly different between two groups (<1.3 vs. 5.3 log IU/mL).

**Table 1:** Baseline characteristics of study cohorts.

		Case	Control	*P*
Sex, n (%)	Male	100 (100)	100 (100)	1
Age (Years)	Mean ± SD	53.8±6.5	53.3±7.2	0.583
ALT (U/L)	Mean ± SD	14.7±10.5	116.9±292.1	<0.001
Serum HBsAg level at baseline (log IU/mL)	Mean ± SD	<−1.3	3.2±1.1	
Serum HBV DNA level (IU/mL), n (%)	>20	0 (0)	95 (95)	<0.001
	<20	100 (100)	5 (5)	
Genotype, n (%)	B	10 (10)	77 (77)	
	C	10 (10)	20 (20)	
	B+C	3 (3)	3 (3)	
rs3077, n (%)	AA	9 (9.0)	4 (4.0)	
	GA	33 (33.0)	35 (35.0)	0.359
	GG	58 (58.0)	61 (61.0)	
rs9277535, n (%)	AA	15 (15.0)	8 (8.0)	
	GA	42 (42.0)	34 (34.0)	0.024
	GG	43 (43.0)	58 (58.0)	

### Two SNPs: rs3077 and rs9277535

Both SNPs met the conditions of Hardy-Weinberg equilibrium and were in moderate linkage disequilibrium (LD) (D′ = 0.64 and r^2^ = 0.36). The distribution of rs3077 genotype was similar between case and control group (*P* = 0.666). There was a higher frequency of rs9277535 non-GG genotype in case group (57% vs. 42%). Chronic HBV carriers with rs9277535 non-GG genotype had higher chance to clear HBsAg (OR: 1.83, 95% CI: 1.04∼3.21, P = 0.034) (Table 2). We constructed haplotypes of these 2 SNPs (rs3077 and rs9277535) for association analysis correlating with HBsAg seroclearance. Compare of GG haplotype, GA haplotype was associated with higher chance of HBsAg seroclearance (OR 2.17; 95% CI 1.14∼4.16, P = 0.030) (Table 3).

**Table 2:** Associations of two SNPs (rs3077, rs9277535) with chronic HBV infection and HBsAg seroclearance.

		Case	Control	Odds ratio	*P*
				(95% confidence interval)	
rs3077, n (%)	GG	58 (58.0)	61 (61.0)	1	
	Non-GG (AA & GA)	42 (42.0)	39 (39.0)	1.13 (0.64∼1.99)	0.666
rs9277535, n (%)	GG	43 (43.0)	58 (58.0)	1	
	Non-GG (AA & GA)	57 (57.0)	42 (42.0)	1.83 (1.04∼3.21)	0.034

**Table 3:** Comparison of association between each haplotype of rs3077-rs9277535 and HBsAg seroclearance.

Haplotype	Allele frequency (%)		Odd ratio	*P*
rs3077-rs9277535	Case	Control	(95% confidence interval)	
GG	58.2	69.5	1.00	0.020
GA	16.4	9.0	2.17 (1.14∼4.16)	0.030
AG	5.8	5.5	1.27 (0.51∼3.21)	0.989
AA	19.6	16.0	1.47 (0.86∼2.50)	0.282

## Discussion

HBsAg loss is an important milestone in the natural history of chronic HBV infection. In this study, which focused on male chronic HBV carriers who acquired the infection perinatally, we found that carriers who had rs9277535 non-G/G genotype had a higher chance to clear HBsAg.

A plenty of studies already addressed the clinical significance of HLA-DP SNP on clearing HBsAg [23]–[26] in patients with acute HBV infection. All these reports compared HBV carriers (HBsAg-positive) with subjects who exposed to HBV but cleared HBsAg (HBsAg-negative but anti-HBc-positive). The main concern of such studies is to put patients who cleared HBsAg after being a chronic carrier and those who cleared HBsAg after acute infection together. In fact, the HBsAg clearance rates are totally different considering the age when HBV infection is acquired, and the mechanism may also be different [3]. HBV infection usually persists in patients who acquire the infection perinatally and HBsAg clearance during the follow-up has been shown to be a rare event [8], [11], [13]–[14]; while the infection persists only in 1–5% of patients infected after childhood, which indicates that most of them will clear the virus after acute infection [3]. A Taiwanese pediatric study before mass vaccination showed that the seropositive rates for HBsAg were 5.1% and 10.7% in children at age of <1 year and 1–2 years, respectively, and the rates remained quite constant, around 10–11%, thereafter [27]. These data suggest that most chronic HBV carriers in Taiwan acquired the infection in early life through perinatal transmission. A large-scale population-based survey in Taiwan has shown that, in 2002, the seropositive rate for HBsAg is 13.7%, which is quite similar to the prevalence rate in children; while the prevalence of positive anti-HBc but negative HBsAg is 54.8%, which is much higher than the prevalence of positive HBsAg. The former finding suggested that the chance for becoming a chronic carrier is quite limited if the infection occurs after the childhood, and the latter finding may indicate that most anti-HBc-positive patients in Taiwan are those who clear the virus after acute infection, but not after becoming chronic carriers. If the infection pattern is similar in Asia, we believe that most of prior studies investigating how HBV is cleared after an acute infection, while our study investigated how HBV is cleared 40–50 years after they acquired the infection in early life. Interestingly, although the timing of clearing virus is totally different, we found that SNPs in HLA-DP region are both important in viral clearance.

Few studies have investigated whether host genetic factors affect clearing HBsAg spontaneously in chronic HBV carriers. HLA-DPA1 and HLA-DPB1 are 2 components of MHC class II, which is important for presenting antigen and initiating the immune response [16]. In this study, we explored the association between HLA-DP regions and HBV clearance by studying the possible surrogate SNPs, rs3077 for HLA-DPA1 and rs9277535 for HLA-DPB1. The strong association between these 2 SNPs and HBV persistence after acute infection has been documented and validated in Asian people [15], [23]–[26], providing us the rationale to investigate the impact of these two SNPs on clearing HBsAg in Taiwanese patients with chronic hepatitis B infection. All of previous studies have indicated that G allele of rs9277535 increased the risk of persistent infection of HBV. Although the mechanism is unclear, we may hypothesize that patients carrying G alleles have less immune response against HBV, thus are at higher risk of becoming chronic carriers after acute infection. Interestingly, our study showed that chronic HBV carriers with rs9277535 non-G/G genotype had a higher chance to clear HBsAg, which also collaborates with our hypothesis. In other words, the SNP of HLA-DPB1 is associated with clearing HBV both in acute and chronic HBV infection. It warrants more studies to elucidate the viral epitopes presented by different HLA-DPB1 genotypes and their relationship with different immune response against HBV.

This study had several unique features. HBsAg loss occurs at annual rate of 0.5∼2% in HBeAg-negative patients [8], [11], [13]–[14]. A large cohort with a long-term follow-up period is mandatory to evaluate this rare event. In addition, we have a strict enrollment criterion to ensure that the include patients to fit our requirement, allowing us to understand more about HBsAg loss in Taiwanese HBV carrier. Also, our study had some limitations. First, we usually determine the HBV genotype using a PCR-based method with the lower detection limit of 100 copies/mL. In this study, chronic HBV carriers who cleared HBsAg had HBV DNA levels less than 20 IU/mL (∼100 copies/mL). Therefore, we can not to investigate the interaction between HBV genotype and HBsAg seroclearance in the study. Second, spontaneous HBsAg loss occurs with a low incidence rate. It is not easy to collect a large number of patients, thus the statistical power may not be strong enough to fully address this issue. Although limitations exist in this study, our data still provide new light in the spontaneous HBsAg seroclearance in chronic hepatitis B patient.

In summary, in consistent with the relationship between HLA-DP SNP and HBsAg clearance after acute HBV infection, we found rs9277535 non-GG genotypes in HLA-DPB1 region are associated with higher chance of spontaneous HBsAg seroclearance in chronic HBV carriers.
